# Findaureus: An open-source application for locating *Staphylococcus aureus* in fluorescence-labelled infected bone tissue slices

**DOI:** 10.1371/journal.pone.0296854

**Published:** 2024-01-31

**Authors:** Shibarjun Mandal, Astrid Tannert, Bettina Löffler, Ute Neugebauer, Luís Bastião Silva

**Affiliations:** 1 Leibniz-Institute of Photonic Technology (Member of Leibniz Health Technologies, Member of the Leibniz Centre for Photonics in Infection Research, LPI), Jena, Germany; 2 Center for Sepsis Control and Care, Jena University Hospital, Jena, Germany; 3 Institute of Medical Microbiology, Jena University Hospital, Jena, Germany; 4 BMD Software, PCI-Creative Science Park, Ílhavo, Portugal; University of Bologna / Romagna Local Health Authority, ITALY

## Abstract

*Staphylococcus aureus* (*S*. *aureus*) is a facultative pathogenic bacterium that can cause infections in various tissue types in humans. Fluorescence imaging techniques have been employed to visualize the bacteria in ex-vivo samples mostly in research, aiding in the understanding of the etiology of the pathogen. However, the multispectral images generated from fluorescence microscopes are complex, making it difficult to locate bacteria across image files, especially in consecutive planes with different imaging depths. To address this issue, we present Findaureus, an open-source application specifically designed to locate and extract critical information about bacteria, especially *S*. *aureus*. Due to the limited availability of datasets, we tested the application on a dataset comprising fluorescence-labelled infected mouse bone tissue images, achieving an accuracy of 95%. We compared Findaureus with other state-of-the-art image analysis tools and found that it performed better, given its specificity toward bacteria localization. The proposed approach has the potential to aid in medical research of bone infections and can be extended to other tissue and bacteria types in the future.

## Introduction

**Fig 1:**
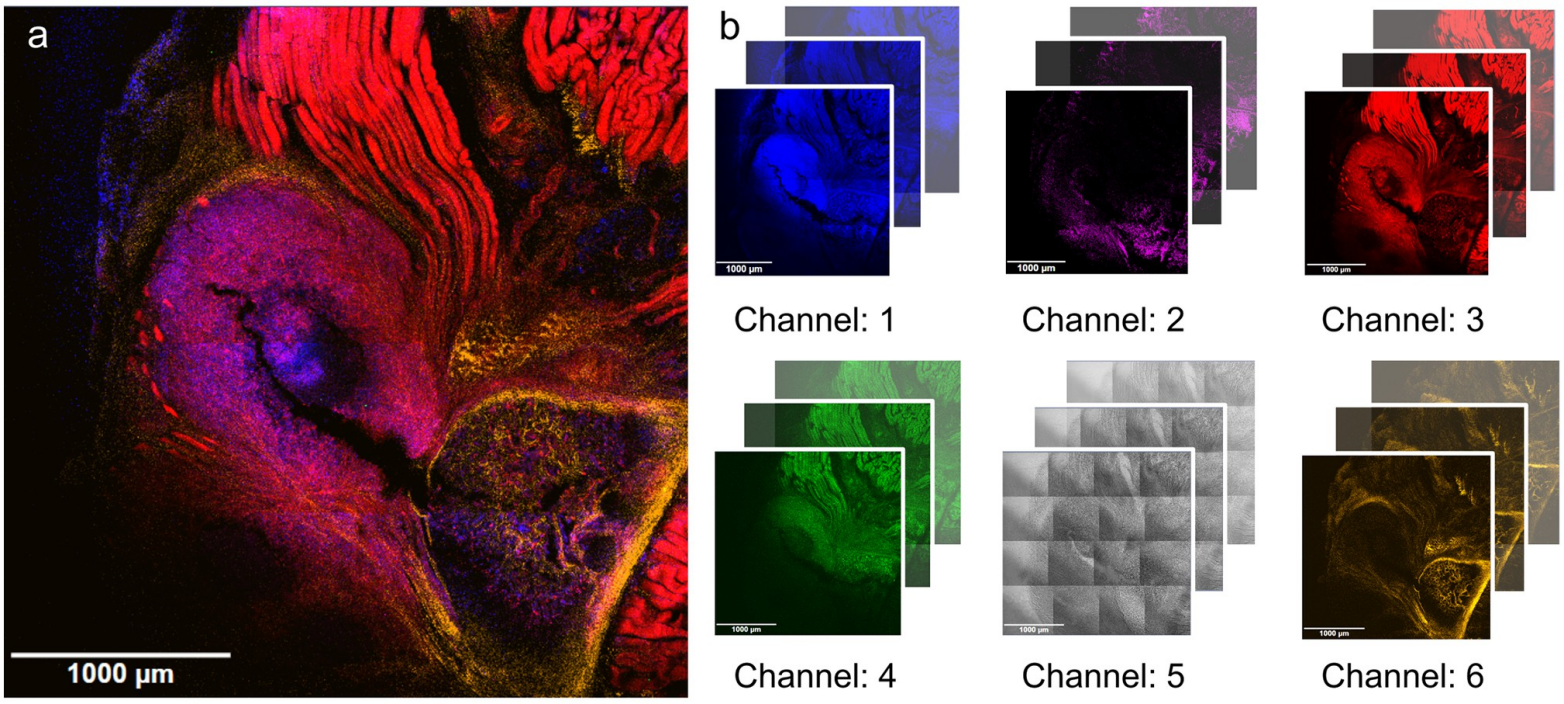
Typical CLSM multispectral image of an infected mouse bone tissue. 3788 pixels (x) × 3788 pixels (y) × 3 (z) × 6 (channel). (a) Overview image scan of an infected tissue at z plane 2, with all fluorescence channels merged, the brightfield channel was omitted. (b) Illustration of the different channels: channel 1: nuclei, channel 2: osteocalcin, channel 3: actin cytoskeleton, channel 4: *S*. *aureus*, channel 5: brightfield, channel 6: collagen. Details regarding tissue staining and image acquisition can be found here [3].

Visualization of pathogens in tissue sections using fluorescence microscopy, e.g. after immunofluorescence labelling in disease models, is a growing tool to gain further insight into pathogenesis and to develop treatment options. This approach is particularly significant in the context of *Staphylococcus aureus* (*S*. *aureus*), the bacterium responsible for various human infections [1, 2], affecting different tissues. It involves the use of various dyes and labeled antibodies to illuminate specific cells and structures, resulting in the generation of intricate multispectral images, which can be either two-dimensional (2D) or three-dimensional (3D) in nature (refer to Fig 1).

The quality and accuracy of acquired images can be influenced by factors such as fluorophore choice and instrumentation setup, leading to variations in pixel intensity and noise sources like photon counting noise, camera readout noise, and tissue auto-fluorescence, which can introduce inaccuracies [4]. Researchers typically rely on software from instrument vendors or compatible scientific image viewers to visualize and locate bacteria, with options ranging from free open-source tools to licensed or subscription-based software. Enhancing intensity measurements for bacterial regions often involves adjusting image brightness and contrast through histogram review, though this approach may be subjective and impractical for extensive analysis, potentially limiting thoroughness and reliability. In the context of free open-source tools, image analysis software tools like ImageJ [5], CellProfiler [6], Ilastik [7], and Qupath [8] streamline bacteria localization through thresholding, segmentation, and feature extraction, but challenges persist in the predominantly manual thresholding process, and existing automatic methods may lack reliability and time spending. Additionally, in most cases, evaluations often prioritize cellular conditions over complex tissues.

To address the demanding task of localizing bacteria in immunofluorescently labelled tissue slices, this paper introduces Findaureus, an open-source Python-based standalone application. Findaureus is a tool that aims to assist researchers in identifying the presence and location of bacteria in fluorescence images of infected tissue slices. Our application has been tested on infected mouse bone tissue images only, due to limitations in available datasets. However, the design is generic, and with further refinement, it can be extended to cover other tissue types as well. This application is open-source, meaning it is available for free to anyone who wants to use it and is currently compatible with any Windows OS-operated computer. The application aims to simplify the process of analyzing fluorescence images to locate bacteria, by offering user-friendly tools and extracting key information. Moreover, the extracted information is machine-readable and can be easily used by other applications for further research and measurement analysis.

## Methods and materials

### Dataset

The dataset comprises image files of infected tissue obtained from the pelvic bone of a mouse model [9] with chronic hematogenous osteomyelitis induced by *S*. *aureus* infection. To prepare for imaging, the mouse was sacrificed, and its pelvic bone was carefully extracted and decalcified using 14% ethylenediamine tetraacetic acid (EDTA) and 0.2% paraformaldehyde. Subsequently, immunofluorescence labeling was performed to detect *S*. *aureus* on 100 μm thick bone cryosections. Additionally, osteocalcin, nuclei, and cytoskeleton components were subjected to counterstaining, enabling the visualization of the host response, host cell morphology, and their spatial distribution. A total of six infected tissue samples were subjected to scanning using a multiphoton confocal laser scanning microscope at the Jena University Hospital, Germany. Further details regarding the preparation of mouse bone tissue, and the imaging methodology can be found in the linked reference [3].

The mentioned tissue samples were thoroughly scanned with dimensions ranging from 1024x1024 to 13925x14847 pixels and 2–8 channels, taken at z plane depths of 0–54μm (~28 z planes). Furthermore, over 50 detailed scans of specific regions of interest (ROIs) were obtained from the tissue samples, with dimensions from 256x256 to 1316x1316 pixels, and 2–8 channels at z plane depths of 0–100μm (~270 z planes). All images were acquired in CZI format with a total size of approximately 129 GB. Despite the lack of publicly available infected tissue datasets, particularly in the context of bone tissue, which poses limitations for the application of artificial intelligence (AI) and machine learning (ML) in our bacteria localization application, the statistical model we propose in this article successfully achieved the desired outcomes.

For users’ convenience, the image scans utilized in this article have been made publicly available for individual testing, as outlined in the ’Data availability’ section, and are comprehensively documented in the associated GitHub repository. If you use these data and image files for scientific publication or any other purpose, please acknowledge and cite this article [3], as they are exclusive to this research publication.

### Modules overview

We needed an application that is user-friendly, produces vital information about bacteria, and can be reused further as needed. Furthermore, we desired a standalone application that could function on any Windows operating system without any dependency software and could swiftly load high-resolution images. As confocal fluorescence images can be quite large and necessitate substantial processing power and time for analysis, a standalone application can effectively utilize the resources of the local computer, leading to quicker results.

Findaureus was developed using Python 3.9.7 and several open-source Python packages, including:

a) AICSImageIO v4.11.0 [10]: package for reading metadata and imaging data of the input fluorescence image file. It supports most bio-formats from Open Microscopy Environment (OME).
b) czifile v2019.7.2 [11]: package for reading metadata and imaging data from input fluorescence image files with the CZI extension, which is the native file format used by the ZEN software developed by Carl Zeiss Microscopy GmbH
c) Webcolors v1.3 [12]: package for working with channel color codes and color names
d) OpenCV-Python v4.7.0.72 [13]: package for managing morphological operations
e) NumPy v1.24.3 [14]: package for performing operations for image processing and analysis
f) Matplotlib v3.6.2 [15]: plot signal/image at scale and present an interactive visualization of images
g) PySimpleGUI v4.60.5 [16]: package for creating cross-platform user-friendly graphical user interfaces (GUIs).

The application consists of two packages: one with the necessary functions to run the application and GUI, and the other containing validation tools and functions for validating the algorithm. The executable file (Findaureus v1.0.0) can be downloaded separately from the GitHub repository’s ‘Releases’ section. The validation tools are not intended for frequent use by end-users, their main purpose is to provide a benchmark for validating the algorithm’s performance and accuracy. This allows for ongoing improvements and implementation of new algorithms.

### Image analysis workflow

In this workflow, the application employs image analysis techniques that commence by loading and scrutinizing the image file’s metadata to acquire significant image details, including the number of accessible channels, image size in pixels, scaling per pixel, scaled image size, and number of z planes. The information obtained is used to create image arrays with precise dimensions and navigate through different z planes (if more than one exists in the image file). To extract the image arrays that belong to the bacteria channel, it is necessary to manually select the channel, since it varies depending on the user’s preference during image acquisition. The resulting 2D image array/s are of unsigned 8-bit integers (uint8) with or without z planes (depending on the acquisition). The algorithm used for detecting bacteria is optimized for 8-bit image data types. Therefore, the arrays are checked and converted to 8-bit before being processed further.

**Fig 2:**
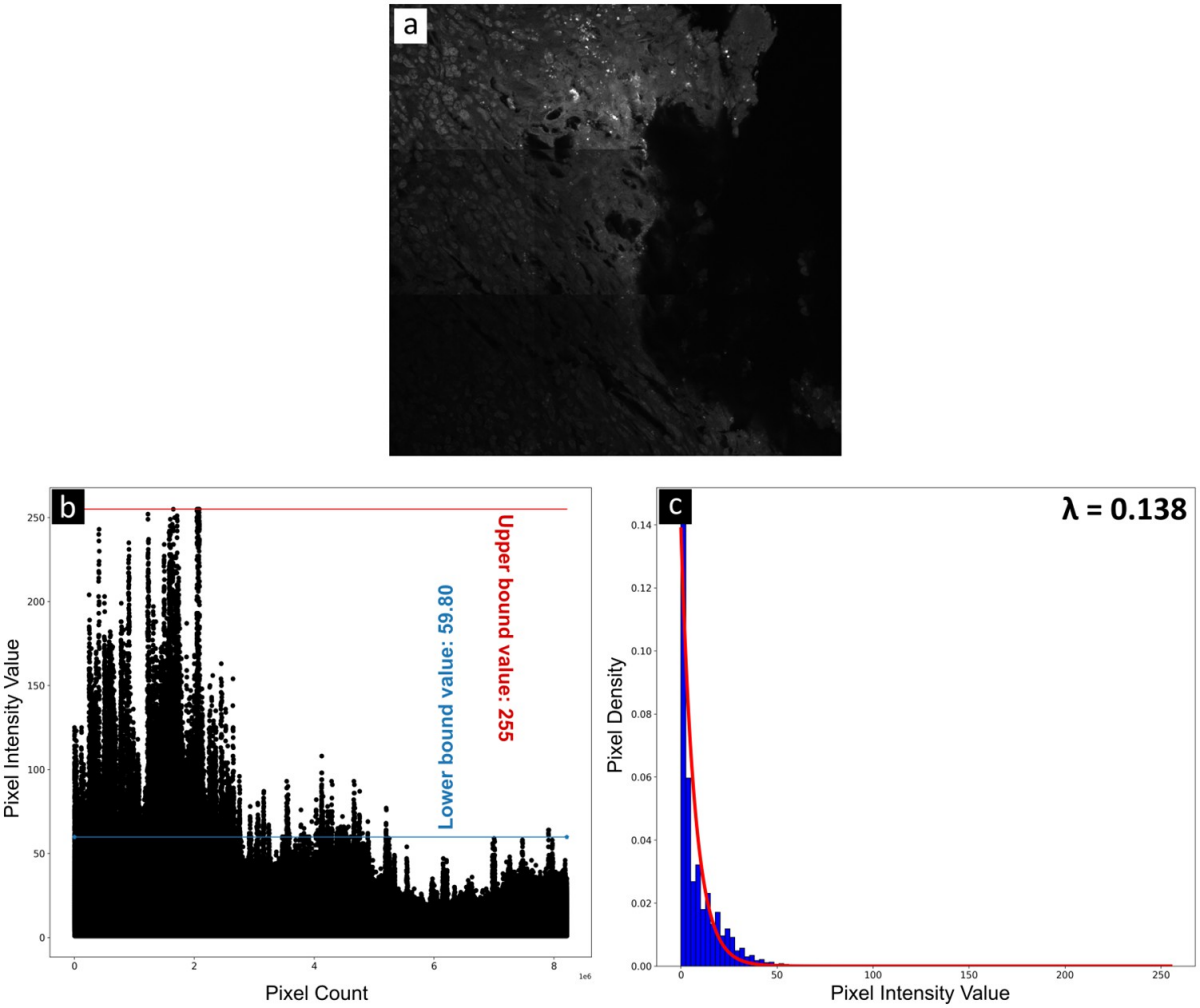
Histogram and pixel scatter plot of the input image array demonstrating the prevalence of background pixels and bacterial pixels. (a) Input image. (b) Pixel scatter plot of the input image. (c) Histogram of the input image with the exponential distribution (in red) based on the calculated lambda (inset).

Examining an input image (Fig 2a), a noteworthy observation emerges when we analyze the scatter plot (Fig 2b) of the input image array pixel intensities. In this plot, it becomes apparent that the majority of pixels are associated with lower intensity values, representing background or noise. The remaining pixels with higher intensity values, correspond to the bacterial region. This phenomenon occurs because the interaction between the photons emitted from the laser and the fluorophore (in this case, bound to bacteria) results in higher pixel intensity values within the image array. The scatter plot not only displays the inherent uncertainty surrounding the presence and distribution of bacteria in tissue samples but also illustrates substantial interference from background elements, often occurring amidst competing cellular structures and sources of noise like autofluorescence or nuclear staining of cell nuclei. While our algorithm is adept at effectively locating bacteria in such complex images, it is important to clarify that it is not intended to handle cases where bacteria are already highly pronounced and dominant in the images.

To separate only the bacterial region, we create a dynamic range-thresholding operation on the input image array using a higher bound value and a lower bound value. As shown in the scattered plot (Fig 2b), the upper bound value will always be the maximum pixel value in the input image array. To obtain the lower bound value, we calculate the quantile value of exponentially distributed pixel intensities from the histogram of the input image array (Fig 2c). The majority of the histograms of the images in our dataset follow the exponential distribution pattern. Additional histograms and scatter plots of pixel intensities of the images used in the "Results and discussion" section can be found in the supplementary material, specifically, S1 Fig. This lower bound value helps us differentiate the bacterial region from the background by considering a user input probability of how many pixels could be in the background and how many could be in the bacterial region. In our situation, we consider a high probability of background pixel occurrence, as evident by both the histogram and the scatter plot of the input image array. Consequently, any pixel value in the image array that is less than or equal to the quantile value is considered background, while any pixel value that is higher than the quantile value is part of the bacterial region.

The quantile value of the exponential distribution for a probability p, where 0 ≤ p < 1 can be expressed as

$$quantile=\frac{-\text{ln}\left(1-p\right)}{\lambda }$$ (1)

λ is the rate parameter of the exponential distribution, which can be estimated using the median value using the median formula of exponential distribution i.e. ($median=\frac{\text{ln}\;2}{\lambda }$), represented as

$$\lambda =\frac{\text{ln}2}{median\;of\;the\;image's\;pixel\;intensity\;array}$$ (2)

From empirical evidence observed throughout the dataset, the probability of background pixel occurrence (p) was considered as 0.999, i.e. 99.9% of the pixel belong to the background and only 0.1% belong to the bacterial region.

Substituting the obtained values (λ, p) in the quantile formula (1), the equation can be written as

$$quantile=\frac{-\text{ln}\left(1-0.999\right)}{\text{ln}2}\times median\;of\;the\;image's\;pixel\;intensity\;array$$ (3)

The upper and lower bound value (quantile) considered for the input image (Fig 2a) is also highlighted in the scatter plot Fig 2b.

**Fig 3:**
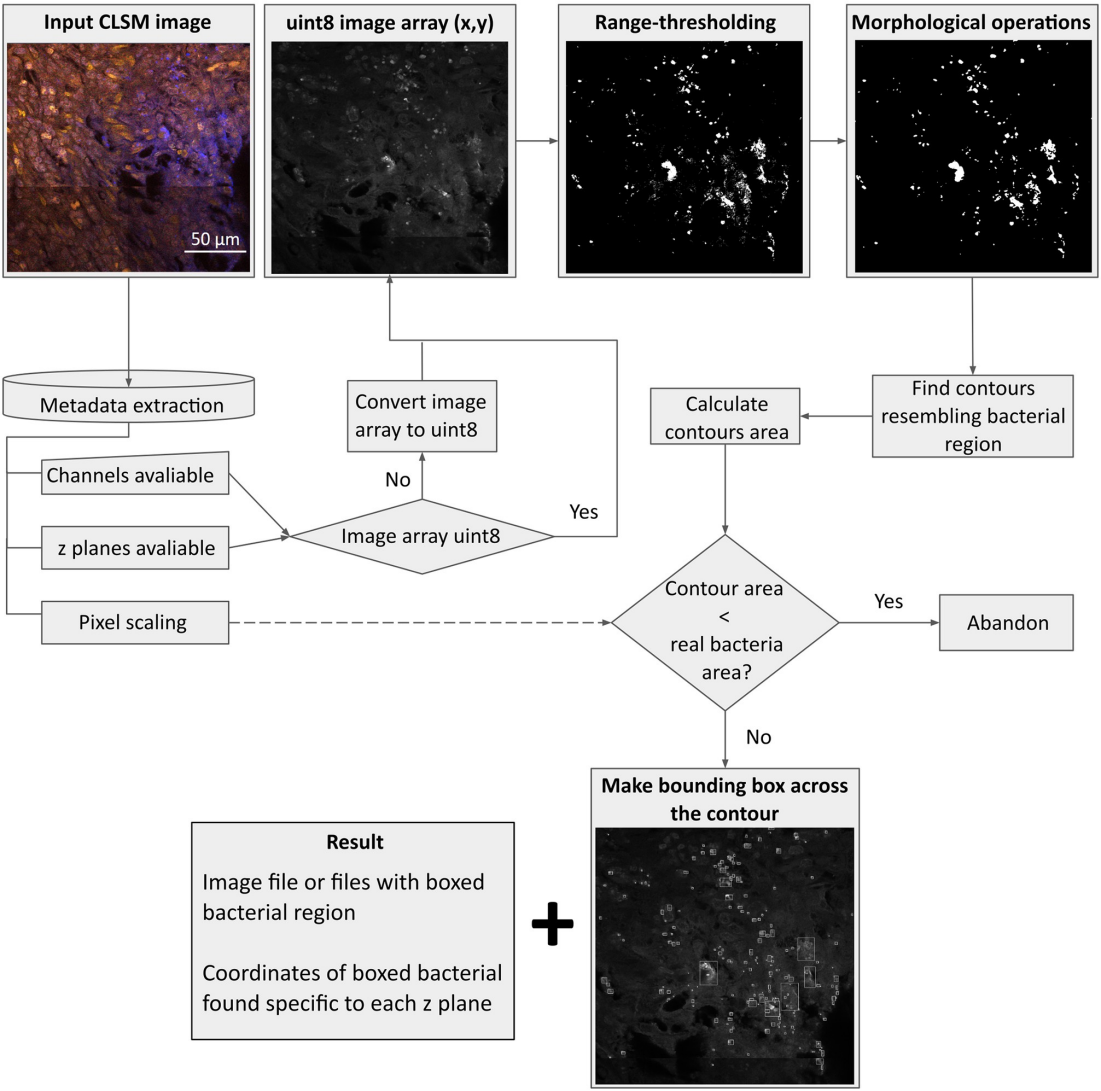
Image analysis workflow.

Even with the range-thresholding operation applied, some regions in the image array contain noise that appears as foreground. To rectify this issue, we implemented two steps. First, we used the OpenCV opening operation, a morphological operation that involves first eroding an image to remove small bright regions, followed by dilating the result to restore the remaining bright regions’ original size and shape. Second, we considered the fact that one *S*. *aureus* bacterium is approximately 0.5–1.5 micrometers in diameter [17], and discarded any areas that were smaller than the smallest bacterium. The bacterium/bacteria areas were calculated from the pixel-wise scaling information in the metadata of the file. In addition, we used connected components analysis to create operational labels defining bacterial regions and created bounding boxes around these regions based on contour vectors. The centroid of the bounding boxes containing the bacterial region was then stored as bacterial coordinates. All the calculated coordinates can be exported in a JSON format and machine-readable format, which can be used easily for further research. Fig 3 displays a flowchart that concisely illustrates the sequence of steps involved in the application’s workflow.

### Graphical user interface (GUI)

**Fig 4:**
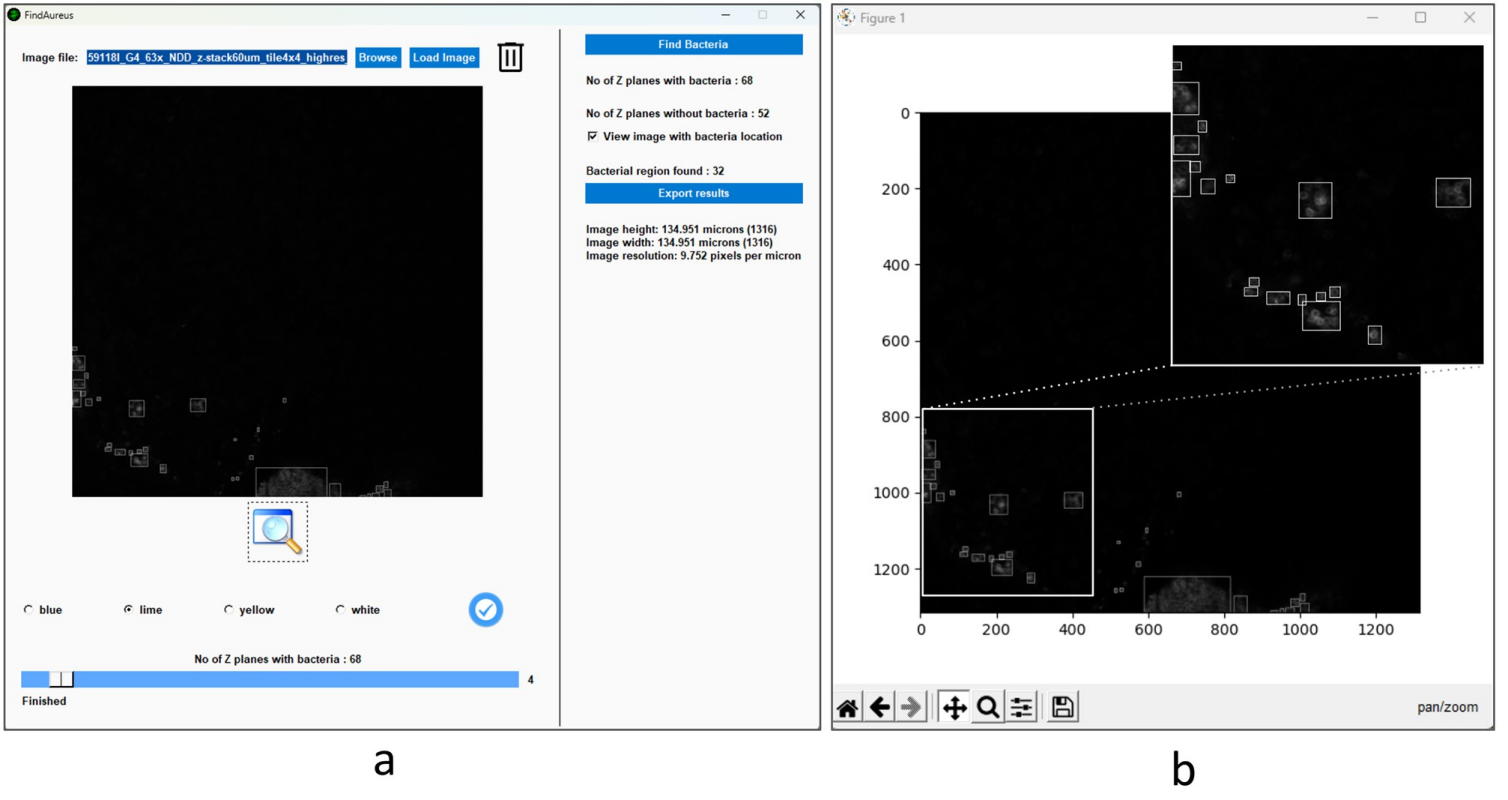
Illustrates the user-friendly features available within the application. (a) shows an overview of the application, highlighting the various features that are available to the user. (b) shows the features that are available within the explore icon, including options for zooming, panning, editing the plot, and saving the figure.

The application’s GUI is supported by the PySimpleGUI python library, which wraps tkinter, Qt (pyside2), wxPython, and Remi (for browser support)—the most popular libraries for handling the GUI. We designed this application interface and its features with input from researchers, students, and scientists who work on a daily basis in the fields of fluorescence microscopy and bone infection. We took into consideration their needs and requirements when creating this interface, intending to create a tool that would be useful and effective for them in their work. Fig 4a shows an overview of Findaureus, the features available are pointed out in the following:

1. Slider bar: Allows you to easily navigate through different z planes, with the option to display only those with bacteria present.
2. Explore icon: Provides the ability to examine the current image in more detail and interactively, including features such as zooming, panning, editing the plot, and saving the figure, as shown in Fig 4b.
3. Information on the number of z planes with and without bacteria: Gives you an overview of the distribution of bacteria in the images.
4. Toggle button ’view image with bacteria location’: Enables or disables bounding boxes around the bacterial regions in the image viewer and updates the slider bar to show only those z planes with bacteria present.
5. Information on the bacterial regions found in the current image: allows to easily understand the distribution of bacteria in the image.
6. Information on the image size in pixels and microns, including resolution.

Findaureus is currently available for Windows operating systems as an executable file, which means that there is no need to install any dependencies separately. Once the application is downloaded, it is ready to use.

### Benchmarking with synthetic images

To assess the performance of Findaureus, we employed synthetic images of infected mouse bone tissues. These images were generated for validation and benchmarking by artificially introducing bacteria into images initially devoid of any bacterial presence. The details of these methods will be elaborated upon in the following.

We created an augmented bacterial image dataset containing bacterial regions by extracting multiple segments from our collection of infected tissue images, with varying pixel intensities. Further, we augmented this bacterial region by shuffling pixel intensities, altering the size, flipping, and blurring the images. The augmented bacterial image dataset is available on our GitHub repository.

Next, we identified image files from our dataset that contained a sufficient amount (10–200) of z plane images with non-bacterial regions. Findaureus, as mentioned in Table 1, can discern z plane images lacking any bacterial presence. On these images with only background, we added random numbers of bacterial regions from the augmented bacterial image dataset with random coordinates and recorded these coordinates as our “Actual Bacteria”, ground truth for bacterial regions. The resolution of the bacteria in the images differed based on the type of scan performed. To account for this, we recorded the pixel scaling from the metadata for each image before generating the artificial infected tissue images. This allowed us to ensure that the size of the simulated bacterial regions in the images accurately reflects the size of the bacteria as observed in the original images. Apart from the pixel coordinates representing bacteria, we treated all other coordinates on the image array as "Actual Background," which signifies the background.

**Table 1:** Relevant details of the input fluorescence image files used in Fig 5 and identified bacterial regions by the proposed method.

	No. of z planes available	No. of z planes with bacteria	No. of z planes without bacteria	Average no. of bacterial regions found across z planes
**Image 1**	120	104	16	3.47
**Image 2**	1	1	0	1195
**Image 3**	96	94	2	143.28
**Image 4**	120	86	34	23.69

## Results and discussion

**Fig 5:**
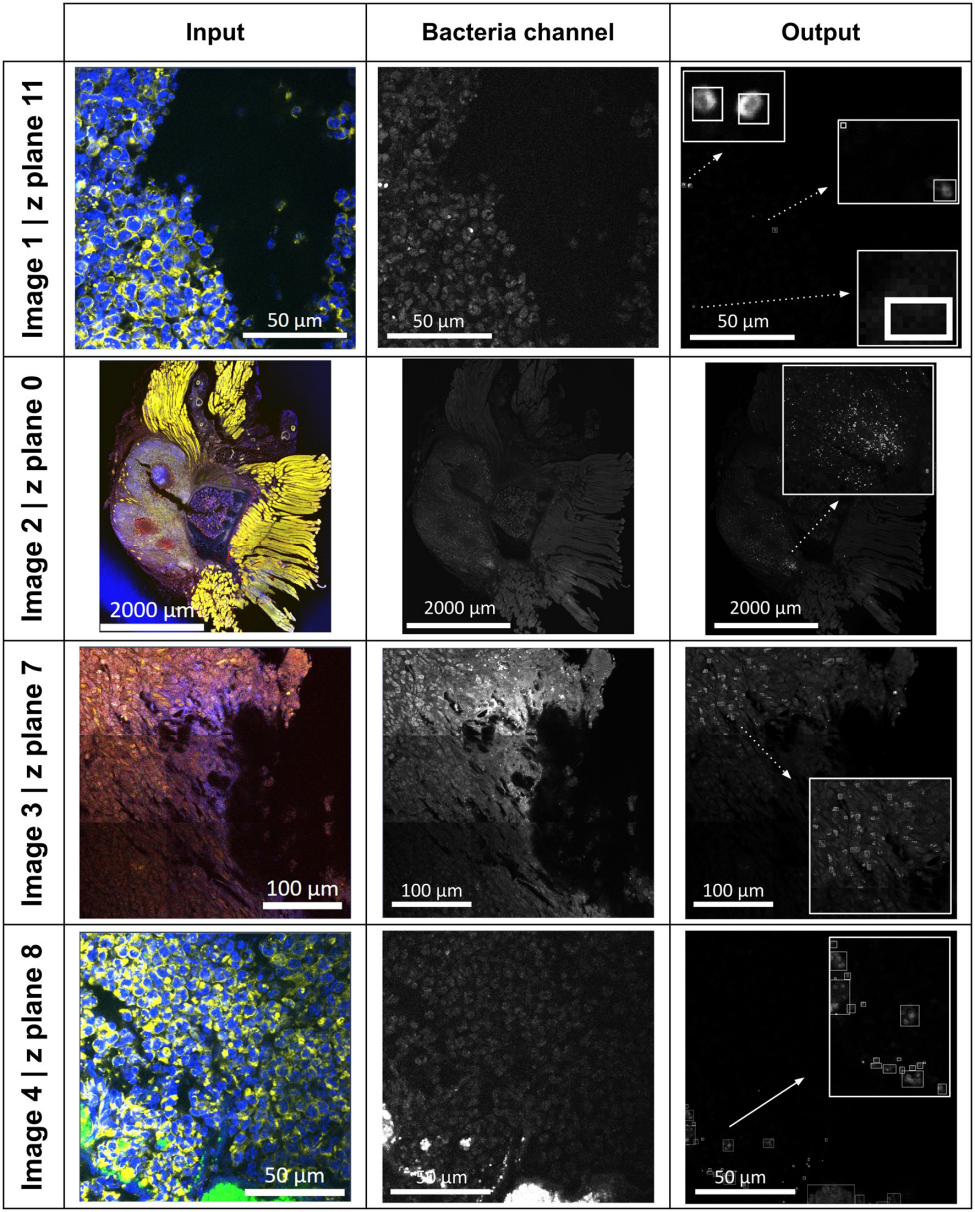
Examples of the results obtained using the proposed algorithm to identify bacterial regions in infected mouse bone tissue. Inset-boxed bacterial regions were highlighted with arrows in the ‘Output’ column. Image 2 is reprinted from [3] under a CC BY license, with permission from MDPI, original copyright 2023.

The application performs well with the datasets listed in the “Dataset” section, and some of the results are shown in Fig 5 with their relevant details in Table 1. The histogram fitting and the scatter plot of pixel intensities of the images can be found in the supplementary materials figure, S1 Fig. Image 1, 3, and 4 shown in Fig 5 represents detailed tissue ROI image scan with a dimension ranging from 1316–2866 pixels (*x*) × 1316–2866 pixels (*y*) × 96–120 (z) × 4 (channel), whereas Image 2 represents overview tissue image scan with a dimension of 10750 pixels (*x*) × 12696 pixels (*y*) × 0 (z) × 5(channel).

In the context of automating bacterial localization, prior research has primarily emphasized segmentation methodologies and the identification of fluorescent spots, which serve to represent individual or clustered fluorophores indicative of bacterial entities [18, 19]. Substantial advancements have been made in the development of specialized software tools dedicated to the analysis of bacterial images, encompassing both open-source solutions such as ImageJ [5], CellProfiler [6], Ilastik [7], QuPath [8], Colicoords [20], and eHooke [21], as well as commercial packages like Matlab [22], MetaMorph [23], and Huygens [24]. While these tools offer a broad spectrum of image processing capabilities, they may not possess specific features tailored exclusively for bacterial analysis. Furthermore, the datasets commonly employed to assess the performance of these applications are frequently optimized for controlled cellular conditions, and all the software programs necessitate a degree of manual intervention during specific preprocessing stages. Despite our exploration of these tools, we encountered challenges related to compatibility and differences in underlying principles. Consequently, a comprehensive review was conducted, taking into account the specific parameters and outcomes relevant to our task, wherein it was observed that Findaureus meets all the criteria for bacterial identification, seamlessly aligning with our specific requirements (Table 2). The subsequent table provides a summarized comparison between Findaureus and other freely available software tools.

**Table 2:** Comparison of available free software tools for automatic bacteria localization with Findaureus.

	ImageJ/Fiji [5]	CellProfiler [6]	Illastik [7]	QuPath [8]	Colicoords [20]	Ehooke [21]	Findaureus
**Programming language**	Java	Python	Python	Java/Groovy	Python	Python	Python
**Supported formats**	OME Bio-Format, DICOM, HDF5	OME Bio-Formats, TIF, or PNG is recommended	PNG, JPG, TIF, HDF5	OME Bio-Formats, OpenSlide	TIF	TIF	CZI, ND2, LIF, OME Bio-Formats
**Stand-alone application**	Yes	Yes	Yes	Yes	No	No	Yes
**Bacterium-specific**	No	No	No	No	Yes (rod-shaped)	Yes (spherical shaped)	Yes (*S*. *aureus*)
**User-intervention**	Yes	Yes	Yes	Yes	Yes	Yes	No
Adaptive or manual thresholding	Yes	Yes	Yes	Yes	Yes	Yes	No
Annotations required	No	Yes	Yes	Yes	Yes	Yes	No

**Fig 6:**
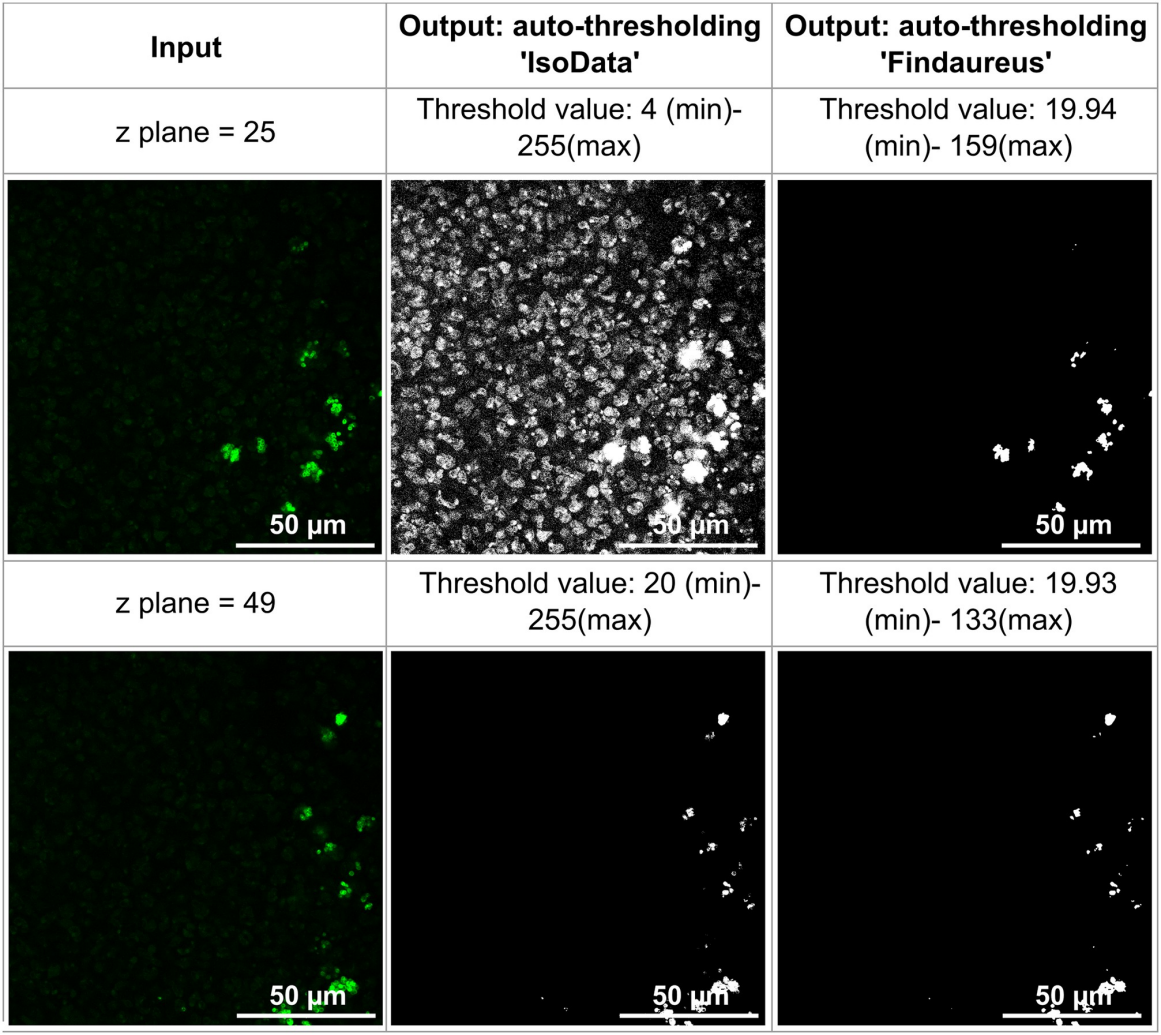
Inconsistency was observed in the auto-thresholding algorithm ’IsoData’ provided by ImageJ, further results were compared with our auto-thresholding Findaureus operation. The conventional method thresholded both bacterial and non-bacterial regions, while the proposed method accurately thresholded only the bacterial region in both the z planes.

It is worth mentioning that the proposed method is not suitable for standard cultivation-based methods, which provide information about the number and type of bacteria by destroying the tissue samples. Our method, on the other hand, addresses the complexity of localizing bacteria (fluorescently labelled) in intact tissue samples. We aimed to identify effective methods for bacterial localization within our infected mouse bone tissue images using various image analysis software, including vendor-provided solutions. Many software packages offer a macro utility for automating image processing steps, but this approach proved challenging due to significant image variability across z planes and different image files. Notably, ImageJ [5], a widely-used biological image analysis software, provides an ’Analyze particles’ feature allowing users to set a particle size threshold for bacterial identification. However, this method often identifies non-bacterial regions like noise, and its performance is inconsistent due to variations in image characteristics. Fig 6 illustrates the application of the ’IsoData’ auto-thresholding method to a single image file from the dataset, revealing the challenges posed by non-uniform images across different z planes. Given the specific nature of our task, such inconsistencies were expected.

### Validation

**Fig 7:**
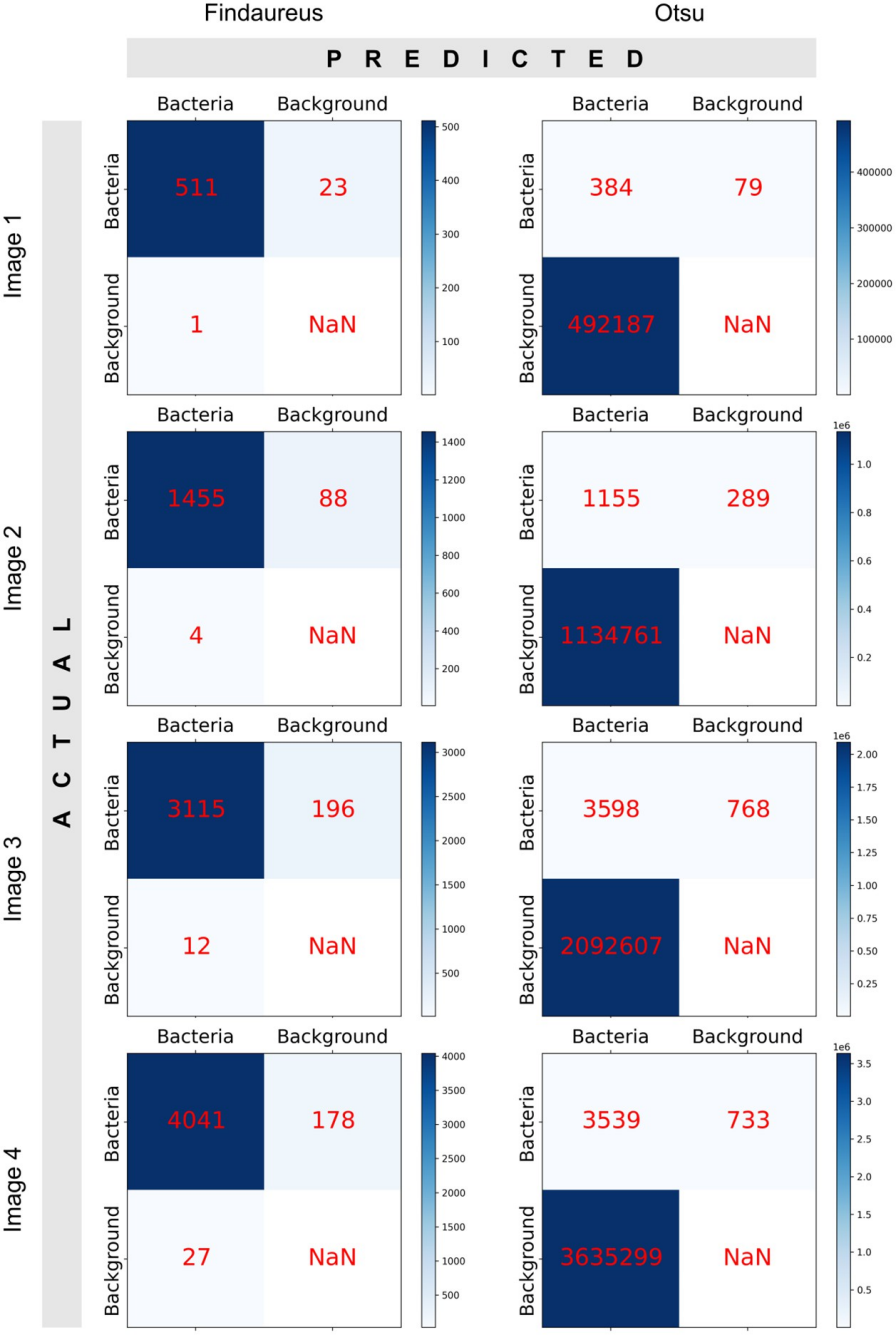
Comparison of confusion matrices between Findaureus and Otsu’s thresholding method findings, as presented in Table 3.

With the approach presented in the subsection “Benchmarking with synthetic images” in the “Materials and methods” section, we conducted the statistical analysis, which includes calculating accuracy, precision, sensitivity, and F1 score. We have shown the relevant equations in the supplementary section (S1-S4 Eq in S1 File). In addition, respective confusion matrices were also generated and presented in Fig 7. It is important to note that in our statistical analysis, we did not explicitly consider or assess background information such as TN (True Negatives) because the current version of the Findaureus algorithm is focused solely on identifying bacterial regions and doesn’t provide predictions for background regions. The results of our analysis are presented in Table 3.

**Table 3:** Performance comparison of Findaures and Otsu’s thresholding method on simulated infected mouse bone tissue images (Accuracy, Precision, Sensitivity, and F1 Score) across four trials.

CLSM image file	No. of z planes with Bacteria	Method	Accuracy (%)	Precision (0–1)	Sensitivity (0–1)	F1 Score (0–1)
**Image 1**	12	**Findaureus**	96.34 ± 1.15	1.00 ± 0.00	0.96 ± 0.01	0.98 ± 0.01
		**Otsu**	64.87 ± 2.27	0.77 ± 0.02	0.83 ± 0.06	0.73 ± 0.00
**Image 2**	38	**Findaureus**	95.08 ± 1.11	1.00 ± 0.00	0.95 ± 0.01	0.97 ± 0.01
		**Otsu**	66.64 ± 4.94	0.82 ± 0.04	0.78 ± 0.04	0.75 ± 0.05
**Image 3**	90	**Findaureus**	94.44 ± 0.81	1.00 ± 0.00	0.95 ± 0.01	0.97 ± 0.01
		**Otsu**	68.81 ± 1.23	0.83 ± 0.02	0.83 ± 0.01	0.77 ± 0.02
**Image 4**	110	**Findaureus**	95.76 ± 0.64	0.99 ± 0.00	0.96 ± 0.00	0.98 ± 0.00
		**Otsu**	70.00 ± 0.36	0.84 ± 0.00	0.84 ± 0.00	0.79 ± 0.00

We tested our Findaureus algorithm for validation on around 250 images (four CZI image files with multiple z planes) in 4 trials since many aspects of the implementation involved randomization. The image files were in dimension 1316 pixels/134.95 μm (*x*) × 1316 pixels/134.95 μm (*y*) × 120-270/44 μm-99 μm (z) × 4(channel). Findaureus achieved an overall accuracy of 95.40%, a precision of 1, a sensitivity of 0.96, and an F1 score of 0.97. It has been observed that in certain cases where two ground truth bacterial regions are positioned closely together, Findaureus incorrectly identifies them as a single bacterial region with a single coordinate. This misidentification has potentially resulted in a 4.6% decrease in accuracy. However, considering that the algorithm still successfully identifies the bacterial region, we believe this discrepancy can be disregarded.

We used the similar validation method and compared our range-thresholding approach with several well-known automatic thresholding methods, like Otsu [25], Li [26], and Triangle [27] as benchmarks. However, except for Otsu, the Li and Triangle methods had very poor results with an overall accuracy of less than 1%. These methods considered a significant portion of the background, hence we didn’t review them in detail in the paper. On the other hand, Otsu’s method had an overall accuracy of 67.6%, (Table 3, column ‘Method: Otsu’). On further examination, we discovered that Otsu’s method is only effective when there are a small number of backgrounds with low pixel intensities and the bacterial regions (chosen randomly) in the image have high pixel intensities, which clearly differentiate the foreground and background. Otsu’s [25] method uses variance to find the threshold value that minimizes the weighted variance between the foreground and background pixels, which was the case in some particular instances.

The validation with benchmark code is available and stated with instructions clearly in our GitHub repository.

## Conclusion

### Impact

The Findaureus application is designed to help researchers quickly locate bacteria in fluorescence images of infected mouse bone tissue without any image preprocessing steps involved. This open-source Python application is user-friendly and provides tools to extract bacteria-relevant information from complex fluorescence images, making it easy for researchers to analyze a large number of images or work with limited resources. It is important to note that, in research, while Findaureus is a useful tool, it does not replace the expertise and judgment of healthcare professionals. It is still important for healthcare professionals to carefully interpret the results of the application and consider other factors. Overall, Findaureus is a valuable resource for research and clinical communities in their efforts to study bone and other infections.

### Limitations and potential development

The potential drawback of Findaureus at present is that it has only been tested on an infected mouse bone tissue dataset with CZI file extension (native to Carl Zeiss microscopes) due to the limited variety of available datasets. However, other microscopic image file extensions Leica (LIF) and Nikon (ND2) have been tested for compatibility. We anticipate that as the application gains wider usage, we will have the opportunity to interact with a broader range of image file extensions through the use of the OME Bio-Formats package for compatibility. Findaureus is not restricted to bone tissue samples, and might as well work for other infected tissue types which were either immunofluorescence labeled or infected with genetically modified auto-fluorescent or bioluminescent bacterial strains. Similarly, since the size of most bacteria is around 1 μm, the application should be also suitable for locating other bacteria than *S*. *aureus*. The users are invited to test their data sets and contact the authors when encountering shortcomings in adapting the algorithm or GUI for their needs. In future developments, Findaureus will focus on improving the accuracy and reliability of the tools and algorithms used for bacterial localization in fluorescence images. Another limitation is the consideration of general standard bacterial structure for *S*. *aureus* when identifying bacterial regions, assuming a diameter of 0.5–1.5 μm. However, literature suggests that bacteria can adapt their shape to survive in specific regions of the bone matrix [28]. In such cases, Findaureus may produce erroneous results. With a larger dataset, it would be possible to incorporate this specific case information and utilize AI techniques to address these variations and improve accuracy in such scenarios. Additionally, expanding the application of Findaureus to different tissue types and bacterial strains would establish a strong foundation for the development of more advanced AI-based tools. This could involve refining existing methods or exploring new approaches using machine learning to address challenges such as image noise, variability in image quality, or the presence of other structures that may interfere with bacterial detection.

## System configuration

Findaures was built and validated on a Dell Latitude 5411 equipped with an Intel(R) Core(TM) i7-10850H processor and 16 GB of RAM. The operating system was Windows 11, and the Python IDE used was Spyder 5.4.3.

## Supporting information

S1 Fig Visualizing pixel distributions in the ’Results’ section. (TIF)

S1 File (DOCX)
