# Supplementary figures and images for: Findaureus: An open-source application for locating Staphylococcus aureus in fluorescence-labelled infected bone tissue slices
Source: PLoS One. 2024 Jan 31;19(1):e0296854. doi: 10.1371/journal.pone.0296854 (PMC10830009; doi:10.1371/journal.pone.0296854)

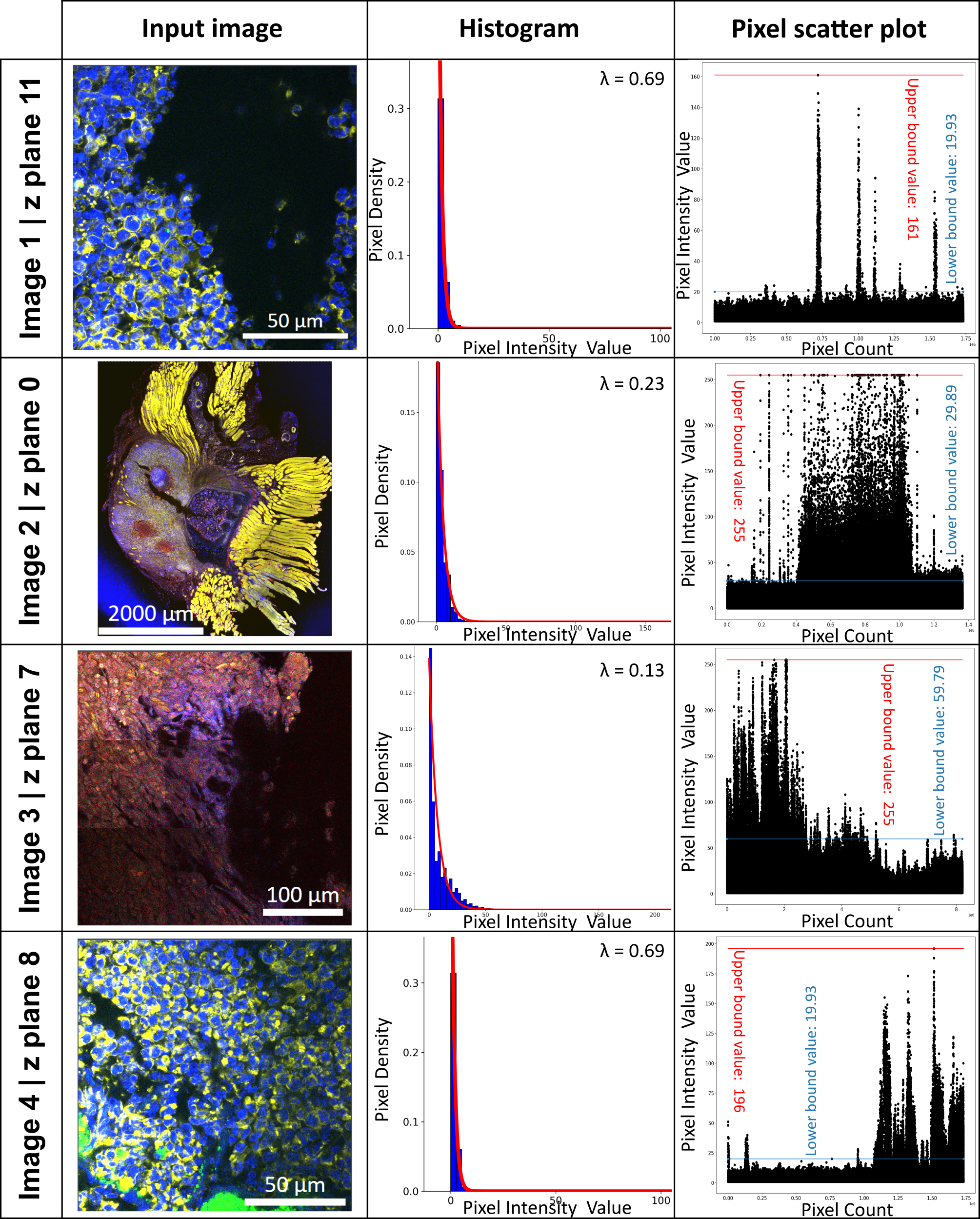

Supplement: S1 Fig — (TIF) [file pone.0296854.s001.tif]
