# Supplementary material for: Findaureus: An open-source application for locating Staphylococcus aureus in fluorescence-labelled infected bone tissue slices
Source: PLoS One. 2024 Jan 31;19(1):e0296854. doi: 10.1371/journal.pone.0296854 (PMC10830009; doi:10.1371/journal.pone.0296854)
Supplement: S1 File — (DOCX) [file pone.0296854.s002.docx]

Findaureus: an open-source application for locating *Staphylococcus aureus* in fluorescence-labelled infected bone tissue slices

Shibarjun Mandal^1^*, Astrid Tannert^1,2^, Bettina Löffler^2,3^, Ute Neugebauer^1,2^ ,Luís Bastião Silva^4^

^1^ Leibniz-Institute of Photonic Technology (Member of Leibniz Health Technologies, Member of the Leibniz Centre for Photonics in Infection Research, LPI), 07745 Jena, Germany

^2^ Center for Sepsis Control and Care, Jena University Hospital, 07747 Jena, Germany

^3^ Institute of Medical Microbiology, Jena University Hospital, 07747 Jena, Germany

^4^ BMD Software, PCI-Creative Science Park, 3830-352 Ílhavo, Portugal

* Corresponding author

E-mail: [Shibarjun.Mandal@leibniz-ipht.de](mailto:Shibarjun.Mandal@leibniz-ipht.de) (SM)

**Contents**

[**Supplementary figures** 3](#_Toc146281492)

[**S1 Fig:** 3](#_Toc146281493)

[**Supplementary equations** 5](#_Toc146281494)

[**S1-S4 Eq** 5](#_Toc146281495)

## **Supplementary figures**


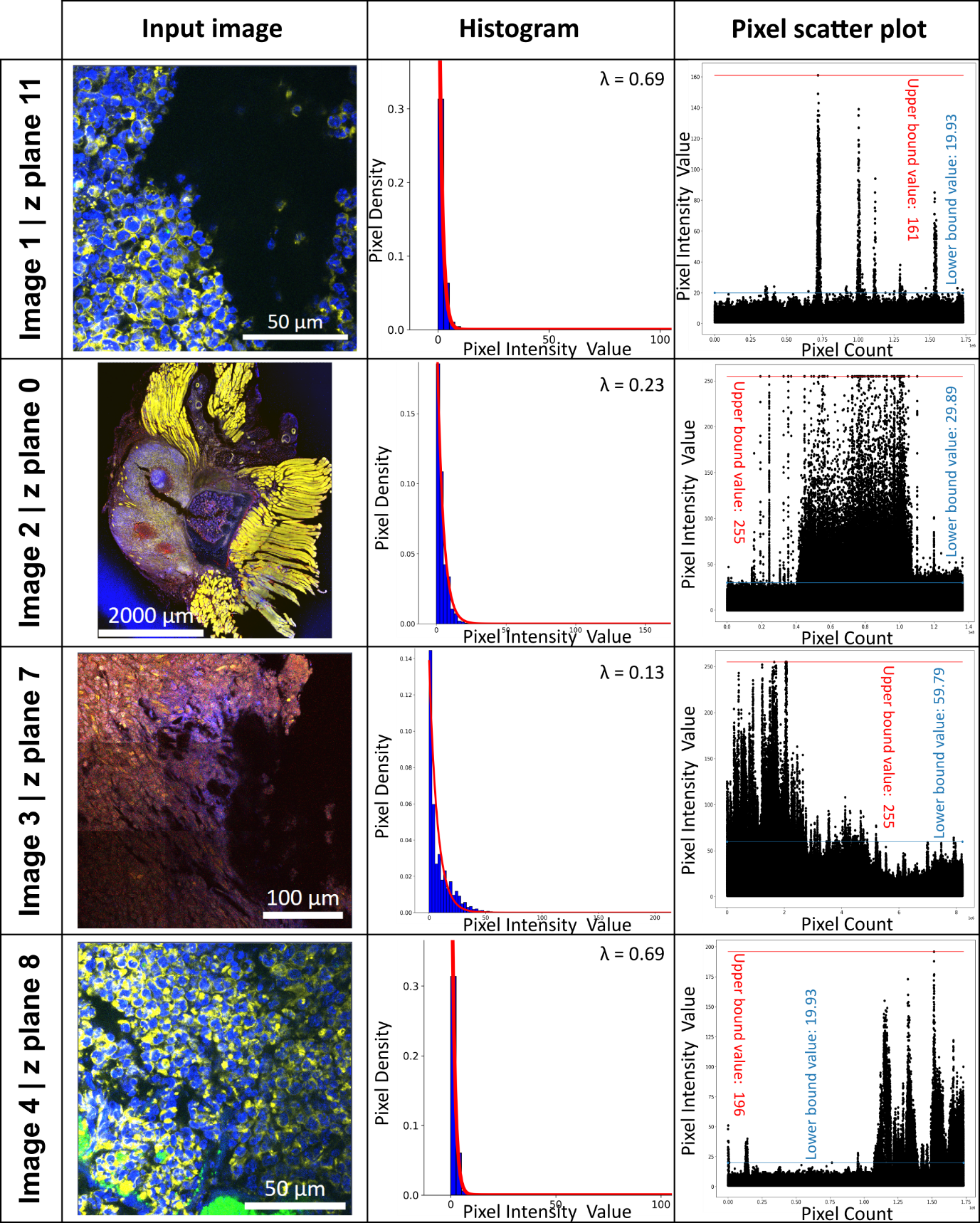


**S1 Fig: Visualizing pixel distributions in the 'Results' section.** Histograms and pixel scatter plots for the input images, illustrating their pixel distributions. The histograms exhibit a consistent exponential distribution (highlighted in red) across our dataset, with the rate parameter specified in the accompanying set. We also include a pixel scatter plot illustrating our auto range-thresholding method, with bounds mentioned in the inset. Image 2 is reprinted from [3] under a CC BY license, with permission from MDPI, original copyright 2023.

## **Supplementary equations**

**S1-S4 Eq: Equation to determine Accuracy, Precision, Sensitivity, F1 score**

$$Accuracy=\frac{True Positives (TP)}{TP+False Positives \left( \mathrm{FP} \right)+False Negatives (FN)}$$

( 1 )

$$Precision=\frac{\mathrm{TP}}{TP+FP}$$

( 2 )

$$Sensitivity=\frac{\mathrm{TP}}{TP+FN}$$

( 3 )

$$F1 score=\frac{Precision\times Sensitivity}{Precision+Sensitivity}$$

( 4 )
